# Revisiting the neuropsychiatry of Huntington's disease

**DOI:** 10.1590/s1980-5764-2016dn1004002

**Published:** 2016

**Authors:** Antonio Lucio Teixeira, Leonardo Cruz de Souza, Natalia Pessoa Rocha, Erin Furr-Stimming, Edward C. Lauterbach

**Affiliations:** 1 Laboratorio Interdisciplinar de Investigação Médica, Faculdade de Medicina, Universidade Federal de Minas Gerais, Belo Horizonte MG, Brazil.; 2 Neuropsychiatry Program, Department of Psychiatry and Behavioral Sciences, McGovern Medical School, University of Texas Health Science Center at Houston, Houston, TX.; 3 Department of Neurology, McGovern Medical School, University of Texas Health Science Center at Houston, Houston, TX.; 4 Department of Psychiatry and Behavioral Sciences, Mercer University School of Medicine, Macon, GA.

**Keywords:** Huntington's disease, neuropsychiatry, cognition, behavior

## Abstract

Huntington's disease (HD) is an autosomal dominant neurodegenerative disease classified under the choreas. Besides motor symptoms, HD is marked by cognitive and behavioral symptoms, impacting patients' functional capacity. The progression of cognitive impairment and neuropsychiatric symptoms occur in parallel with neurodegeneration. The nature of these symptoms is very dynamic, and the major clinical challenges include executive dysfunction, apathy, depression and irritability. Herein, we provide a focused updated review on the cognitive and psychiatric features of HD.

## Abstract (pt)

A doença de Huntington (DH) é uma doença neurodegenerativa autossômica dominante classificada entre as coreias. Além de sintomas motores, a DH é caracterizada por sintomas cognitivos e comportamentais que impactam na capacidade funcional dos pacientes. A progressão dos sintomas neuropsiquiátricos e déficits cognitivos ocorre paralelamente à neurodegeneração. A natureza desses sintomas é muito dinâmica, sendo que os desafios clínicos mais comuns incluem disfunção executiva, apatia, depressão e irritabilidade. O presente artigo apresenta uma revisão atualizada sobre as manifestações cognitivas e psiquiátricas da DH.

## INTRODUCTION

Huntington's disease (HD) is an autosomal dominant neurodegenerative disease caused by the expansion of CAG repeats in the exon 1 of the *HTT* gene at the short arm of chromosome four (4p16.9), responsible for the synthesis of the huntingtin protein. The mutant gene seems to confer a toxic gain of function, leading to characteristic neuropathological changes, including accumulation of nuclear and cytoplasmic inclusions of mutant huntingtin in neurons, with prominent degeneration of the caudate and putamen.^1^

HD is traditionally classified under the choreas, *i.e.* involuntary movement disorders characterized by non-rhythmic and random muscle contractions that can affect any part of the body.^2^ Before the advent of genetic testing, HD was diagnosed by the presence of chorea in the context of a family history of the disease. Indeed, chorea is one the earliest motor manifestations of typical HD. However, chorea is a poor marker of disease progression, stabilizing and/or fading away in late stages of HD. Moreover, patients with early-onset HD, *i.e.* before 21 years of age, tend to develop other motor symptoms, and may not have chorea.^3^ These symptoms include bradykinesia, rigidity, dystonia and motor incoordination.

Besides motor symptoms, HD is marked by cognitive and behavioral symptoms. Non-chorea motor and cognitive changes present with a steady progression, impacting patients' functional capacity. Psychiatric symptoms are also very common, but have a more unpredictable course, sometimes flaring or remitting unexpectedly.^4^ The objective of the current manuscript is to provide a focused updated review on the cognitive and psychiatric features of HD.

## COGNITIVE IMPAIRMENT

The degenerative nature of HD leads to gradually progressive cognitive impairment. Due to the relative preservation of memory at disease onset, cognitive decline in HD has been classically characterized as the "subcortical type".^5^ The term "subcortical dementia" was proposed by Albert in 1974 to refer to the cognitive impairment observed in patients with progressive supranuclear palsy, characterized by executive deficits (mainly slow processing speed and attentional disorders) and behavioral changes (such as apathy), without any evidence of "true cortical" involvement (aphasia, apraxia, agnosia).^6,7^ Since Albert's initial proposal, "subcortical dementia" has been used to describe the cognitive profile of other neurological disorders, including Parkinson's disease and HD. Although this concept is still employed to assign the neuropsychiatric disorders of HD, the concept of "subcortical dementia" has been challenged. Indeed, the advent of modern neuroimaging techniques and new methods of neuropsychological assessment have demonstrated evidence of "cortical" deficits due to subcortical lesions, in the absence of cortical damage.^8,9^ More specifically, it has been increasingly recognized that the striatum has a prominent role in the modulation of complex cognitive abilities in different neurological diseases, including HD.^10^

Executive deficits are the main neuropsychological features of HD, even at pre-manifest and stages of the disease.^10,11^ Patients typically have attentional complaints that are generally referred as "memory disorders". The cognitive exam discloses low performance in working memory, flexibility and planning.^11-14^ These executive deficits are more severe than those observed in Alzheimer's disease at the same disease duration. Adaptive behavior and rule learning are also frequently impaired in HD. In line with these aforementioned difficulties, patients with HD generally have low performance on the Wisconsin Card Sorting Test, Trail Making Test and Spans. There is also impairment in planning and problem-solving abilities, as demonstrated by difficulties on the London Tower test.^11-14^

Memory complaints are frequent among HD patients. Besides complaints, an objective memory deficit is regularly demonstrated in HD, characterized by a low immediate and delayed recall performance on both verbal and visual memory tests. It seems that episodic memory impairment in HD is mainly due to a retrieval deficit, due to an impaired ability to activate strategies needed for retrieving stored information.^15-17^ This recall impairment may result from registration and/or retrieval deficits linked to subcortical-frontal dysfunction, rather than from a storage problem due to damage in medial-temporal lobe structures, as seen in Alzheimer's disease. Indeed, HD and Alzheimer's disease differ in their episodic memory profile. While there are recall deficits in both diseases, patients with HD benefit from semantic cueing, which is not the case for patients with Alzheimer's disease. Recall performance in HD is generally improved, or even normalized, with the use of semantic cueing.^15,16^ Recognition is also usually preserved in HD, while it is impaired in Alzheimer's disease.

Language impairment is present in HD, with reduced lexical fluency being the most reported finding. Patients with early stage HD may have difficulties in rule application in different domains, such as verbal conjugation and syntax comprehension.^18^ A Brazilian study investigated 23 patients with HD and found low performance on repetition, oral agility and comprehension in both oral and reading modalities.^19^ Besides language deficits, communication abilities of HD patients can be also impaired by dysarthria and involuntary oromandibular movements.

More recently, it has been demonstrated that social cognition is affected in HD. For instance, the identification of negative facial emotions (anger, disgust, sadness and fear) is compromised early in HD.^20^ There is also emerging evidence that patients with HD have deficits in sarcasm detection and in both affective and cognitive aspects of theory of mind, even at pre-manifest stages of the disease.^21-23^

Taken together, the recent advances in the neuropsychological investigation of HD have demonstrated evidence of cognitive deficits beyond those assigned to the concept of "subcortical dementia".

It is worth emphasizing the dynamic nature of cognitive changes in HD. Moreover, the rates of cognitive decline seem to differ with disease progression. The rate of change tends to accelerate with disease progression, particularly after the critical point of moving from undiagnosed to diagnosed HD.^24^ From a neurobiological standpoint, it has been proposed that these changes may be the clinical correlate of the activation of intracellular cascades or pathways beyond the accumulation of misfolded huntingtin.^25^

Neuroimaging studies have shown a robust correlation between cognitive performance and imaging parameters. For instance, caudate volume significantly correlated with different executive function tests in patients with HD.^26^ Using diffusion tensor imaging, Delmaire et al. (2013) reported correlation between mean diffusivity in the anterior part of the putamen and the caudate nucleus, and executive functioning as assessed by the Trail Making Test, Part B.^27^ It is important to mention that imaging measures, notably striatal atrophy, have shown the largest effect size for tracking HD progression so far.^28^

Higher cognitive reserve was significantly associated with a slower rate of change in executive function (Trail Making Test, Part B) and slower rate of volume loss in caudate and putamen for HD mutation carriers estimated to be closest to motor symptom onset, *i.e.* 'phenoconversion'.^29^ However, cognitive reserve modified the rate of cognitive decline only in patients at phenoconversion, and not among those HD mutation carriers estimated to be distant from motor onset. These results corroborate the hypothesis of disease acceleration at the time of 'phenoconversion', and striatal atrophy as a pivotal biomarker in HD.

## BEHAVIORAL OR NEUROPSYCHIATRIC SYMPTOMS

Systematic studies on behavioral and psychopathological issues of HD were scarce until the 2000s when a growing volume of literature emerged. Older studies reported that personality changes were found in 70% of patients with HD,^30^ but most of these studies lacked reliable diagnostic tools, and did not follow current classification systems (i.e. the Diagnostic and Statistical Manual of Mental Disorders or the International Classification of Disorders).^5^ The relationship between CAG trinucleotide repeat length and psychiatric manifestations of HD has also been investigated in relatively small samples with contradicting results.^31,32^

It is misleading to regard behavioral and/or psychiatric symptoms in HD as a homogeneous and/or static entity. Independent groups identified clusters of behavioral symptoms in HD. Based on the Problem Behaviors Assessment in HD, three clusters of symptoms were identified: apathy, depression, and irritability.^33,34^ Using data from the European Huntington's Disease Network REGISTRY that assessed the Unified HD Rating Scale in almost 2,000 HD mutation carriers, Rickards et al. (2011) identified four distinct behavioral patterns: depression, irritability/aggression, psychosis, and dysexecutive.^35^ The latter included apathy, obsessive and compulsive behaviors. The Unified HD Rating Scale covers 11 psychiatric symptoms, including anxiety, depressed mood, irritability, perseveration, apathy, delusions and hallucinations that are individually scored according to their severity and frequency.^35^

Based on the analysis of the same REGISTRY database, it was reported that less than 30% of mutation carriers had no neuropsychiatric symptoms in the month prior to study evaluation.^36^ Apathy was reported by 47.4% of subjects, while depression and irritability/aggression occurred in 42.1% and 38.6%, respectively. Moderate-to-severe apathy affected 28.1% of subjects, while moderate-to-severe depression occurred in 12.7%. Obsessive-compulsive behaviors and psychosis occurred in 25.8% and 4.1% of subjects, respectively. These neuropsychiatric symptoms were present in all stages of HD, but tended to be more frequent in late or advanced stages of the disease. Among the five neuropsychiatric syndromes assessed in the study, *i.e.* depression, irritability/aggression, psychosis, apathy, and obsessive-compulsive behaviors, apathy showed the strongest association with disease progression. The prevalence of moderate-to-severe apathy increased with disease stage from 11% in stage 1 to 54.6% in stages 4 and 5.36

Important lessons can be extracted from the REGISTRY study:

i) neuropsychiatric syndromes are very common in HD, already in the pre-manifest stage, but not all patients exhibit severe symptoms;
ii) the prevalence of neuropsychiatric syndromes tend to increase with disease progression;
iii) apathy is the best behavioral or neuropsychiatric correlate of disease progression in HD.

More recently, the PREDICT-HD study, that followed a cohort of over 1,000 HD mutation carriers for up to ten years, confirmed that neuropsychiatric manifestations develop more often than previously thought in the HD pre-manifest stage, and increase with progression of disease severity.^37^ Other studies, including the longitudinal study TRACK-HD, confirmed apathy as an early manifestation and a core neuropsychiatric feature of HD that correlated with its progression.^24,38^ Interestingly, apathy is also appointed as the best behavioral predictor of Alzheimer's disease progression.^39^

Depressive disorders are the second-most-common neuropsychiatric problem in HD. In clinical practice, the best discriminators of depression in HD are affective symptoms (*i.e.* sadness, loss of interest, guilt and suicidality) in contrast with somatic symptoms, such as impairment of appetite, sleep, and psychomotor retardation, that may overlap non-psychiatric problems.^35^ Factors potentially associated with depression in HD include female sex, and a positive history of depression as the main correlate in the REGISTRY study (Odds ratio, OR = 5.57). It is tempting to speculate whether this positive psychiatric history for depression represents very early symptoms related to HD pathological changes instead of a traditional risk factor. Besides this, the role played by psychosocial factors in HD associated depression remains to be better defined. Depressed mood is a very important predictor of suicidal ideation that affects approximately 10% of HD mutation carriers.^40^ Older studies reported that 25% of patients had at least one suicide attempt throughout the disease, and suicide was the ultimate cause of death in up to 10% of patients.^41^

Older studies also reported schizophrenia-like psychosis in a significant number of patients with HD.^42^ Psychiatrists even believed that a patient with schizophrenia-like psychosis might later be diagnosed with HD.^5^ In fact, psychotic disorders occur in few patients with HD, with a prevalence of below 5%.^36^ Psychosis usually develops in more advanced phases of disease and/or in individuals with a longer duration of disease.

Irritability and aggression are frequent behavioral symptoms in HD. In the REGISTRY and TRACK-HD studies, these symptoms were also associated with functional decline in HD, but to a lesser extent than apathy.^24,36^ Accordingly, along with apathy, they can be regarded as signs of frontal lobe dysfunction in HD. Other behavioral problems, such as drug abuse, sleep disorders and hyper-sexuality, can be of clinical concern in HD.

## TREATMENT

Based on the multiple pathophysiological mechanisms implicated in HD, different pharmacological strategies have been evaluated in pre-clinical and clinical studies in HD.^28,43,44^ However, to date there are no disease-modifying strategies available for HD. Promising strategies in pre-clinical models were not confirmed in clinical trials. For instance, latrepirdine, a molecule originally developed as an oral antihistaminic and later shown to stabilize mitochondrial membranes and function, failed to influence cognition and global function in a randomized, double-blind, placebo-controlled trial comprising 403 patients with mild to moderate HD.^45^ Further molecules acting on mitochondrial function or oxidative stress (e.g. coenzyme Q10), and other biological processes such as neuroinflammation (minocycline) did not indicate evidence of efficacy in halting or slowing down HD progression.^46,47^ There is a great hope that genetic-based interventions, such as RNA interference and antisense oligonucleotides, will change this scenario.^44^

In clinical practice, treatment is directed to target symptoms and/or syndromes, such as chorea, irritability/aggression, depression and cognitive decline. In its guidelines for the management of chorea in HD, the American Academy of Neurology recommends the use of tetrabenazine, amantadine and riluzole.^48^ However, tetrabenazine, a specific inhibitor of vesicular monoamine transporter (VMAT2), is the only FDA-drug approved for chorea in patients with HD.^49,50^ There is also preclinical evidence of neuroprotective effects of tetrabenazine in a HD model.^51^ The adverse effects with tetrabenazine include somnolence, depressed mood, agitation and akathisia. Considering the high frequency of depressive symptoms in HD and their potential exacerbation with tetrabenazine, patients taking this drug must be closely monitored for depression and suicidal ideation. In this latter context, amantadine and riluzole could be better therapeutic options.

Behavioral and cognitive interventions are warranted for managing neuropsychiatric and cognitive symptoms, but their evidence of efficacy is very limited.^52,53^ Pharmacological strategies can improve symptom management, but again formal evidence is lacking. Treatment recommendations for neuropsychiatric symptoms are based on expert opinion surveys, open label and/or small controlled studies.^53-55^ Therefore, this is an area with urgent need of research, especially taking into consideration that some strategies may have broad therapeutic and/or deleterious potentials. The antidepressants, for instance, may be theoretically effective in the treatment of mood symptoms, and there is evidence of prevention of neurodegenerative changes characteristic of HD, awaiting clinical confirmation.^56^ Conversely, antipsychotics or anti-dopaminergic drugs that can be used for the treatment of psychotic phenomena, irritability/agitation or even chorea, may lead to more advanced and rapidly progressing HD.^57^

Small controlled studies with atomoxetine, donepezil, and rivastigmine, and open trials with memantine did not find positive effects of these drugs on the cognition of patients with HD.^58-62^ Recently, a 26-week, randomized, double-blind, placebo-controlled trial with 109 early-stage to mid-stage HD patients did not show improvement of a composite of cognitive measures in patients assigned to PBT2, a compound that might reduce metal-induced aggregation of mutant huntingtin.^63^ However, compared with the placebo group, the Trail Making Test Part B score improved between baseline and 26 weeks in the PBT2 250 mg group, but not in the 100 mg group. This potential benefit in executive function must be confirmed in independent studies. It must also be evaluated whether this cognitive improvement translates into better global functioning.

## CONCLUSION

Neuropsychiatric and cognitive symptoms are integral components of the clinical spectrum of HD, reflecting pathological changes in the striatum and cortical regions. In parallel with neurodegeneration, there is progression of cognitive impairment and neuropsychiatric symptoms, mainly apathy and irritability/aggression that can be regarded as signs of frontal lobe dysfunction.

There are no disease-modifying strategies for HD, and current treatment is directed to target symptoms, especially motor and behavioral symptoms. The available evidence for the treatment of neuropsychiatric and cognitive manifestations in HD is very limited, and studies to date have failed to show convincing results. Accordingly, there is a large venue for therapeutic investigation in HD.
